# Outcomes of Patients With Mild Cognitive Impairment With Lewy Bodies or Alzheimer Disease at 3 and 5 Years After Diagnosis

**DOI:** 10.1212/WNL.0000000000209499

**Published:** 2024-06-13

**Authors:** Calum A. Hamilton, Paul C. Donaghy, Rory Durcan, Joanna Ciafone, Kirsty Olsen, Gemma Roberts, Michael J. Firbank, Louise M. Allan, John-Paul Taylor, John T. O'Brien, Alan J. Thomas

**Affiliations:** From the Translational and Clinical Research Institute (C.A.H., P.C.D., R.D., J.C., K.O., G.R., M.J.F., J.-P.T., A.J.T.), Newcastle University; Centre for Research in Ageing and Cognitive Health (L.M.A.), University of Exeter; and Department of Psychiatry (J.T.O.B.), School of Clinical Medicine, University of Cambridge, United Kingdom.

## Abstract

**Background and Objectives:**

Retrospective studies indicate that dementia with Lewy bodies (DLB) may be preceded by a mild cognitive impairment (MCI) prodrome. Research criteria for the prospective identification of MCI with Lewy bodies (MCI-LB) have been developed. We aimed to assess the prognosis of a prospectively identified MCI-LB cohort at 2 key milestones, 3- and 5 years after diagnosis, to examine classification stability over time and rates of adverse outcomes (dementia or death).

**Methods:**

This was a retrospective examination of data from 2 longitudinal observational cohort studies where participants with MCI were prospectively recruited from North East England and differentially classified as MCI due to Alzheimer disease (MCI-AD), possible MCI-LB, or probable MCI-LB. Adverse outcomes (DLB/other dementia or death) and stability of disease-specific classifications were examined in each group.

**Results:**

Of 152 participants with baseline MCI (54 MCI-AD, 29 possible MCI-LB, and 69 probable MCI-LB), 126 were followed for up to 3 years (mean age 75.3 years; 40% female). We found that prospective probable MCI-LB classifications were both sensitive (91%) and specific (94%) to classifications either remaining as probable MCI-LB or progressing to DLB (in some cases autopsy confirmed) for 3 or more years after. Classifications were at least as stable as those in MCI-AD. In this cohort with disease-specific MCI classifications, rates of progression to dementia were high: 55% of MCI-LB had developed DLB within 3 years. Dementia occurred in 47% of MCI-AD over the same duration (odds ratio 1.68, 95% CI 0.66–4.26, *p* = 0.278). Premature death was a common competing risk, occurring in 9% of MCI-AD and 11% of MCI-LB within 3 years.

**Discussion:**

These findings support that prospectively identified probable MCI-LB is a prodromal presentation of DLB and that disease-specific classifications of MCI may reliably identify different prodromal dementias.

## Introduction

Dementia with Lewy bodies (DLB) is a relatively common, but under-recognized form of dementia.^1^ Research criteria have described the prodromal presentation of DLB in people with mild cognitive impairment (MCI): MCI with Lewy bodies (MCI-LB),^2^ and a recent systematic review demonstrated the evidence supporting this concept.^3^ MCI has been described as a prodromal manifestation of subsequent DLB in several cohorts, providing a strong justification for this theorized staging.^4-7^ However, these studies have typically either not classified MCI-LB specifically^5^ or retrospectively classified this based on eventual clinical or pathologic DLB diagnosis.^4,6,7^

Published criteria for MCI-LB have recommended that the predictive validity of these criteria be demonstrated in prospective studies before being applied in clinical settings.^2^ This would demonstrate that prospectively identified MCI-LB is itself a stable classification, which reliably develops into DLB.

The predictive validity is particularly important in MCI because the reported prevalence of nondegenerative etiologies, such as functional cognitive disorders, means the progression of this condition can be variable.^8^ As a result, many individuals diagnosed with MCI in community or even specialist settings may never progress to dementia. It is, therefore, crucial to demonstrate that the prospective identification of MCI-LB will not lead to more false positives and resulting iatrogenic harm.

The short-term prognosis of MCI-LB, has been previously described, with a more rapid development of dementia in individuals with MCI with more DLB diagnostic features.^9,10^ These prospective cohorts of MCI-LB are now reaching sufficient maturity to also draw conclusions about the longer term prognosis at clinically meaningful milestones.

We, therefore, aimed to examine 2 key outcomes of prospectively identified MCI-LB at 2 key landmarks of 3- and 5 years after baseline: stability of MCI-LB etiological classifications (whether MCI-LB diagnoses remain as MCI-LB or DLB) and clinical progression from MCI-LB to DLB or death (the proportion that progress to these outcomes). We compared these with the same outcomes observed in a parallel group of MCI due to Alzheimer disease (MCI-AD).

## Methods

### Participants

Participants were drawn from 2 previously reported study cohorts of MCI cases recruited from North East England between 2013 and 2019.^11,12^

They were identified after diagnosis of MCI in local health care services including old age psychiatry, memory services, neurology, or older person's medicine. Participants were required to be 60 years or older and medically stable at baseline, before being screened for inclusion. Exclusion criteria included Parkinson disease for >12 months before onset of MCI, suspected parkinsonism not due to Lewy body disease (i.e., corticobasal syndrome, progressive supranuclear palsy [PSP], or multiple system atrophy), evidence of dementia at screening, a suspected frontotemporal or cerebrovascular etiology, or subjective cognitive decline only without evidence of objective impairment.

Where possible, an informant was sought (caregiver, family member, or close friend), to provide additional information on behalf of the participant.

### Design and Procedure

Both cohorts were assessed in longitudinal studies, with detailed baseline cognitive assessment, clinical interview, and diagnostic imaging taking place over 3 to 5 sessions between 1 and 2 weeks apart. Longitudinal follow-ups were conducted at approximately 1-year intervals initially, with an adaptive schedule during 2020 because of coronavirus disease 2019–related interruptions. Follow-up cognitive assessments and clinical interview were completed in a single visit.

### Cognitive Assessment

Cognitive function was assessed at baseline and follow-up with a comprehensive battery previously described.^11-13^ The Addenbrooke's Cognitive Examination—Revised (ACE-R) acted as the primary measure of global cognitive function, and provided domain-specific supplementary measures.

### Clinical Assessment

#### Clinical Interview

Participants and informants underwent detailed clinical research interview to supplement cognitive testing. This semistructured interview provided detailed information on cognitive performance outside of the testing environment, functional independence, co-occurring conditions, and core or supportive features of Lewy body disease.

Standardized questionnaires and assessments were administered to evaluate the 4 core clinical features of Lewy body disease:

- The North East Visual Hallucinations Interview facilitated investigation into the presence of complex visual hallucinations and related visual experiences^14^
- The Clinician Assessment of Fluctuation and Dementia Cognitive Fluctuations Scale examined fluctuating changes in attention and concentration^15,16^
- The Mayo Sleep Questionnaire examined the presence of REM sleep behavior disorder, and other sleep disturbances, for example, sleep apnea^17^
- The Movement Disorders Society Unified Parkinson's Disease Rating Scale—Motor Examination (UPDRS-III) measured motor impairment and parkinsonism^18^

Results of these investigations were evaluated by the diagnostic panel with respect to the whole clinical picture to determine the presence or absence of each core clinical feature for the purpose of differential classification (see below).

Assessment of independent function was guided by administration of the Instrumental Activities of Daily Living scale with informants.^19^ Basic activities of daily living were assessed by clinical interview with the participant. These were interpreted using clinical judgment with reference to the patient's previous function, rather than cutoff scores alone.

### Imaging

Dopaminergic imaging of the striatum was undertaken using ^123^I-N-ω-fluoropropyl-2β-carbomethoxy-3β-(4-iodophenyl)nortropane (FP-CIT) SPECT, with images rated as normal or abnormal by an expert panel of imaging analysts blind to clinical information.

In the second prospective study, ^123^I-metaiodobenzylguanidine (MIBG) cardiac scintigraphy was also administered at baseline. Heart to mediastinum MIBG uptake ratio (HMR) was quantified, and considered as abnormal given an HMR <1.85 (cutoff derived from local cognitively healthy comparator group).^20^

Consensus research criteria support the use of FP-CIT and MIBG imaging as potential biomarkers for MCI-LB.^2^ Imaging results were, therefore, incorporated into final differential classifications alongside clinical features.

Structural MRI was offered to all participants in the second prospective study at baseline.

### Diagnosis

At baseline and after each longitudinal reassessment, each participant received a diagnostic review by a 3-person panel of expert old age psychiatrists (P.C.D., J.P.T., A.J.T.). Participant diagnosis of MCI was ratified according to National Institute on Aging-Alzheimer's Association (NIA-AA) criteria (subjective/objective decline with preserved independence, minimal aids/assistance).^21^

Diagnosis of any-cause dementia was also considered based on NIA-AA criteria, differentiating this from MCI by the presence of a “significant interference in the ability to function at work or in usual daily activities.”^22^ This was determined by clinical interview incorporating information on instrumental and basic activities of daily living, with consideration of the overall clinical picture (e.g., whether impairments were a decline from previous normal, and whether these were attributable to cognitive impairments or other factors). Consistent with current clinical guidelines,^22^ this judgment was not based on cognitive or functional cutoff scores in isolation. At baseline, diagnosis of dementia was cause for study exclusion. Individuals diagnosed with dementia at follow-up were retained for analysis.

Where individuals died before dementia was identified in study assessments, were too unwell to attend or were lost to follow-up, informants were interviewed alone and clinical records examined to determine if they had developed dementia outside the study.

Non-AD and non-LBD aetiologies in MCI were considered in each case, including frontotemporal,^23^ cerebrovascular,^24^ primary mood and psychiatric disorders, subjective-only cognitive disorders, atypical static MCI, and any other non-AD/LBD aetiologies. These were excluded if identified at initial screening as previously reported^11,12^ and not retained for analysis.

When identified at follow-up as new information arose, MCI cases more consistent with frontotemporal dementias (FTDs), vascular cognitive impairments (VCIs) and other non-AD/LBD neurologic diseases were excluded from further follow-up but retained for analysis of diagnostic sensitivity and specificity here. MCI who appeared to recover at follow-up to a subjective-only impairment (SCI) continued in the study for further follow-up and were also retained for analysis of diagnostic change.

Findings from research MRI and any clinical MRI or CT imaging, where available, were used to identify possible cerebrovascular cognitive impairments, but were not used in the primary AD/LBD aetiological classifications (see below).

### Aetiological Classification

After diagnosis of MCI/dementia at each clinical review, participants were differentially classified as either MCI-AD, possible MCI-LB or probable MCI-LB according to consensus clinical and research criteria,^2,21^ respectively:

- MCI-AD met criteria for MCI, with no core clinical features of LBD present, and no abnormal indicative biomarkers.
- Possible MCI-LB met criteria for MCI and either (1) had 1 core clinical feature of LBD with no abnormal indicative biomarkers, or (2) no core clinical features of LBD with 1 or more abnormal biomarker(s).
- Probable MCI-LB met criteria for MCI and either (1) had 1 core clinical feature of LBD with 1 or more abnormal biomarker(s), or (2) had 2 or more core clinical features of LBD with any biomarker results.

Diagnoses were updated as and when new symptoms emerged. If dementia was diagnosed, participants received comparative aetiological diagnosis of dementia due to AD,^22^ or possible/probable DLB.^1^ For the purpose of this analysis, the initial clinical diagnosis was used for group classification, not incorporating any later emergent features because the aim is to examine the trajectory of the different baseline diagnoses.

### Analysis

Two primary analyses were undertaken: the first assessed the presence of dementia within 3 years of baseline. As death is a competing risk, premature deaths were also examined. While the time of deaths were known exactly, the time of dementia onset was not, and could have occurred unseen at any point between observations. To minimize bias in either direction, the onset of dementia was, therefore, treated as occurring at the midway point between observations, consistent with previous comparable work.^9^ Association between initial MCI subtype and outcome at 3 years, was examined using logistic regression adjusting for baseline age and sex. We corrected for bias with the penalized maximum likelihood estimator (Firth's method). Individuals with an unknown outcome at 3 years because of exclusion, withdrawal, or loss to follow-up were excluded from this analysis. The primary analysis concerned a 2-group comparison of a single binary outcome, and so no adjustment for multiple comparisons was necessary. Group differences were considered statistically significant at *p* < 0.05.

The second analysis was a description of the stability of aetiological classifications over the course of longitudinal follow-up. Of the n = 152 total baseline cohort, individuals with at least 3 years of follow-up, with a gold-standard diagnosis before this (autopsy diagnosis where available, or consensus diagnostic panel DLB/AD dementia diagnosis), or with a diagnosis that was cause for exclusion from follow-up before this (e.g., FTD) were eligible for inclusion in this analysis. Those who withdrew before 3 years of follow-up, or who died before this without autopsy assessment, were excluded from primary classification stability analysis but the effects of imputation of missing outcomes for participants without sufficient follow-up were examined with sensitivity analyses.

A subset of the sample had follow-up up to 5 years post baseline, and data from this further cutoff, analyzed in the same way, are also reported.

As a diagnostically uncertain group, possible MCI-LB were not included in statistical comparisons, but are reported alongside the more diagnostically certain groups of MCI-AD and probable MCI-LB for additional context.

### Standard Protocol Approvals, Registrations, and Patient Consents

All prospective participants and informants provided written informed consent before screening. These studies received favorable ethical approval from the National Research Ethics Service Committee North East—Newcastle & North Tyneside 2 (12/NE/0290 and 15/NE/0420), and all study procedures were conducted in accordance with the Declaration of Helsinki.

### Data Availability

Raw data from the cohorts supporting this analysis are available through the Dementias Platform UK data portal (portal.dementiasplatform.uk/). Analytical code and data to reproduce these analyses are available by request to the corresponding author.

## Results

### Disease Progression in Prospectively Identified MCI-AD and MCI-LB

**Figure 1:**
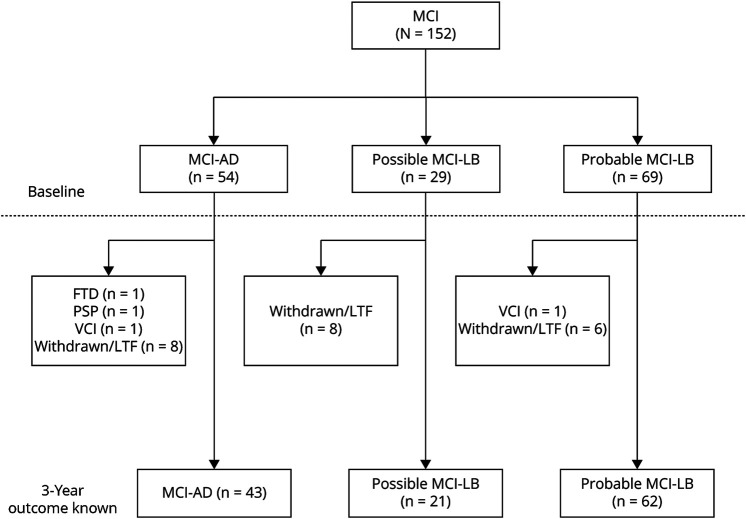
Flowchart for Pooled MCI Cases From Lewypro (n = 75) and SUPErB (n = 77) Cohort Studies, With Data Missingness Because of Withdrawal, Loss to Follow-Up, or Excluded Etiology AD = Alzheimer disease; FTD = frontotemporal dementia; MCI = mild cognitive impairment; MCI-AD = MCI due to AD; MCI-LB = MCI with Lewy bodies; VCI = vascular cognitive impairment.

Of 152 participants initially included, 126 individuals (mean age 75.3 years, 40% female) had sufficient data to include in the primary analysis, with at least 3 years of follow-up, or an adverse outcome (dementia or death) ending follow-up before this. Details of the 26 exclusions are presented in Figure 1, and baseline characteristics of the included group reported in Table 1. Fifty-nine of 62 included MCI-LB cases had undergone FP-CIT imaging, with results being abnormal in 41 (69%). Thirty-four had also undergone MIBG imaging, with results being abnormal in 21 (62%). Ninety-five participants had at least 5 years of follow-up, or dementia/death before this.

**Table 1:** Baseline Participant Characteristics and Clinical Outcome

	MCI subtype (n = 126)		
	AD (N = 43)^a^	Poss. LB (N = 21)^a^	Prob. LB (N = 62)^a^
Age (y)	75.8 (7.4)	74.7 (7.4)	75.2 (7.1)
Female sex	28 (65)	9 (43)	14 (23)
Years in education	12.5 (3.2)	11.4 (3.5)	11.6 (2.8)
ACE-R score/100	81.9 (9.8)	78.9 (12.7)	80.3 (9.1)
MDS UPDRS motor score	14.5 (11.6)	13.8 (9.0)	25.9 (15.7)
Outcome at year 3			
MCI	19 (44)	12 (57)	21 (34)
Dementia	20 (47)	7 (33)	34 (55)
Death	4 (9.3)	2 (9.5)	7 (11)
Outcome at year 5	N = 30	N = 15	N = 50
MCI	3 (10)	2 (13)	8 (16)
Dementia	22 (73)	10 (67)	35 (70)
Death	5 (17)	3 (20)	7 (14)

Abbreviations: ACE-R = Addenbrooke's Cognitive Examination—Revised; AD = Alzheimer disease; LB = Lewy bodies; MCI = mild cognitive impairment.

a Mean (SD); n (%).

Baseline characteristics of the 26 noncompleters were compared with the 126 completers. These groups did not differ significantly in their average age (*p* = 0.36), global cognitive performance on the ACE-R (*p* = 0.96), severity of motor impairment rated by the UPDRS-III (*p* = 0.24), or average level of education (*p* = 0.86).

Outcome by year 3 is reported in Table 1; 20 (47%) cases of MCI-AD had developed dementia by this time with 4 (9.3%) having died before this (crude annualized dementia rates of 16% per year).

Thirty-four (55%) cases of probable MCI-LB had developed dementia, with 7 (11%) deaths before observed dementia (crude annualized dementia rates of 18% per year).

However, with high rates of clinical progression across the cohort, the difference in dementia rates between MCI-LB and MCI-AD at this time point was not statistically significant (odds ratio [OR] 1.68, 95% CI 0.66–4.26, *p* = 0.278). Both adverse outcomes (dementia or death) were common after 3 years, with no significant differences between groups (OR 1.59, 95% CI 0.64–3.91, *p* = 0.314). A Cox proportional hazards survival model incorporating intermediate observations broadly agreed with these findings (MCI-LB hazard ratio 1.36, 95% CI 0.81–2.28, *p* = 0.250).

Increasing age was associated with significantly higher dementia (*p* = 0.021) and overall clinical progression risks (*p* = 0.007): estimated probabilities of remaining alive and dementia free after 3 years were 58% for those aged 60 at baseline, 51% for those aged 70, and 33% for those aged 80.

There was no clear gender-associated difference in dementia prognosis (*p* = 0.925) or overall progression (*p* = 0.856) in MCI overall. Exploratory analyses including a diagnostic group × gender interaction provided weak support for higher dementia progression rates in male patients with MCI-LB compared with MCI-AD (OR 3.70, 95% CI 1.01–13.60, *p* = 0.048), but no significant gender difference in prognosis within MCI-LB (OR 0.17, 95% CI 0.02–1.21, *p* = 0.078).

By year 5, the majority of MCI cases had developed dementia (73% of MCI-AD and 70% of MCI-LB), or died (17% of MCI-AD and 14% of MCI-LB; Table 1).

Sensitivity analyses working on the conservative assumption that those with insufficient follow-up because of withdrawal remained as MCI (last observation carried forward) also agreed with these findings; 44% of MCI-AD and 59% of MCI-LB developed dementia or died by 3 years (crude annualized progression rates of 15% and 20%, respectively), with 50% of MCI-AD and 61% of MCI-LB doing so within 5 years (crude annualized rate of progression 10% and 12%, respectively).

Further sensitivity analyses excluding those clinically classified as SCI based on follow-up information (n = 7) did not meaningfully change any findings.

Patients with MCI who did not progress clinically in this time still demonstrated a significant decline in their global cognitive function (estimated −1.8 [95% CI −2.8 to −0.9] points per year in the ACE-R, *p* < 0.001). However, those who developed dementia or died during follow-up to date had a significantly faster global cognitive decline (estimated −3.5 [−5.0 to −2.0] additional ACE-R points lost per year, *p* < 0.001). Average motor impairment severity increased significantly over the course of follow-up in the MCI group overall (estimated +1.1 [0.4–1.8] points per year in the UPDRS-III, *p* = 0.003) but this did not significantly differ between those who developed dementia/died or remained stable as MCI (*p* = 0.62).

### Aetiological Classification Stability

**Figure 2:**
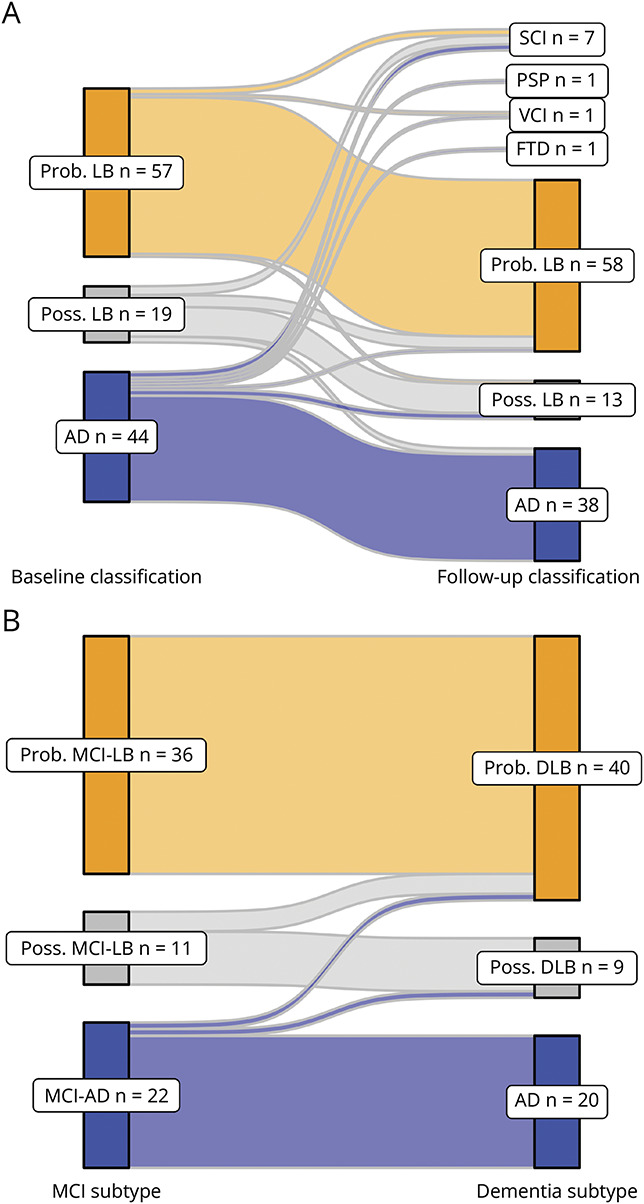
(A) Baseline and 3-Year Follow-Up Classifications (n = 120) and (B) Baseline MCI and Terminal Dementia Classifications, Including Dementia Occurring Beyond 5 Years AD = Alzheimer disease; FTD = frontotemporal dementia; MCI = mild cognitive impairment; MCI-AD = MCI due to AD; MCI-LB = MCI with Lewy bodies; PSP = progressive supranuclear palsy; SCI = subjective-only impairment; VCI = vascular cognitive impairment.

Of the 152 initial MCI cases, 120 were eligible for primary analysis of aetiological classification stability (Table 2 and Figure 2A) with at least 3 years of follow-up, a terminal diagnosis ending study involvement (e.g., FTD), or premature death with autopsy validation.

**Table 2:** Follow-Up Classification Stability in the Full Baseline Sample With Third-Year Definitive Outcome Known (Excluding Death Without Autopsy)

Follow-up classification	Baseline classification (n = 120)		
	AD (N = 44)^a^	Poss. LB (N = 19)^a^	Prob. LB (N = 57)^a^
AD	36 (82)	2 (11)	0 (0)
Poss. LB	2 (4.5)	10 (53)	1 (1.8)
Prob. LB	1 (2.3)	4 (21)	53 (93)
FTD^b^	1 (2.3)	0 (0)	0 (0)
VCI^b^	1 (2.3)	0 (0)	1 (1.8)
PSP^b^	1 (2.3)	0 (0)	0 (0)
SCI	2 (4.5)	3 (16)	2 (3.5)

Abbreviations: AD = Alzheimer disease; FTD = frontotemporal dementia; LB = Lewy bodies; PSP = progressive supranuclear palsy; SCI = subjective-only impairment; VCI = vascular cognitive impairment.

a n (%).

b Excluded from further follow-up.

Of 57 probable MCI-LB cases, 53 (93%) retained a probable MCI-LB or probable DLB classification. One was subsequently classified as possible MCI-LB, 1 VCI, and 2 SCI.

Of 19 possible MCI-LB cases, 10 (53%) remained stable at possible MCI-LB/possible DLB. Four progressed to probable MCI-LB/probable DLB. Two were reclassified as MCI-AD, and 3 as SCI.

Of 44 eligible MCI-AD cases, 36 (82%) retained an MCI-AD or AD dementia classification, with 5 considered to have a non-AD/LBD etiology (VCI, FTD, PSP or SCI), and 3 developing features consistent with possible (n = 2) or probable (n = 1) Lewy body disease.

Within the subset of cases who developed dementia, classifications were substantially more stable (Table 3). In total there were 61 cases of dementia by year 3, 67 by year 5, and an additional 2 cases thereafter, amounting to 69 in total. Of 22 MCI-AD who developed dementia, 20 were diagnosed with AD, and 2 had new symptoms/biomarkers emerge consistent with LBD (1 possible DLB and 1 probable DLB). Of 11 possible MCI-LB with eventual dementia, 8 were classified as possible DLB, and 3 as probable DLB. All 36 probable MCI-LB who developed dementia were classified as probable DLB. Therefore, of 40 prospective cases of probable DLB, 36 (90%) initially appeared as probable MCI-LB (Figure 2B).

**Table 3:** Final Dementia Classifications, Including Dementia Cases Beyond 5-Year Cutoff

Dementia classification	Baseline classification		
	MCI-AD (N = 22)^a^	Poss. MCI-LB (N = 11)^a^	Prob. MCI-LB (N = 36)^a^
AD	20 (91)	0 (0)	0 (0)
Poss. DLB	1 (4.5)	8 (73)	0 (0)
Prob. DLB	1 (4.5)	3 (27)	36 (100)

Abbreviations: AD = Alzheimer disease; DLB = Dementia with Lewy bodies; MCI = mild cognitive impairment; MCI-AD = MCI due to AD; MCI-LB = MCI with Lewy bodies.

a n (%).

### Sensitivity and Specificity

Sensitivity and specificity of MCI-AD and probable MCI-LB baseline classifications were evaluated based on the final known diagnosis for those with sufficient follow-up, or a gold-standard terminal diagnosis before this (autopsy-confirmed, DLB or AD dementia diagnosis, FTD, VCI, PSP or SCI). Five individuals initially seen without probable MCI-LB (1 MCI-AD and 4 possible MCI-LB) were eventually classified as probable MCI-LB/probable DLB at follow-up (false negatives), whereas 53 identified at baseline remained as probable MCI-LB/DLB over repeated follow-up (true positives), indicating 91% sensitivity within this cohort.

Four individuals with baseline diagnoses of probable MCI-LB had this rescinded after follow-up (false positives), while 58 who did not meet baseline criteria for probable MCI-LB did not progress to this over longitudinal follow-up (true negatives), indicating 94% specificity.

While MCI-AD was a somewhat less stable classification overall than probable MCI-LB, in this context the sensitivity was good at 95%, though less specific at 90%.

### Autopsy Validations

To date, 10 individuals who died have donated brain tissue for neuropathologic examination. Four cases had a study classification of MCI-AD, with all 4 (100%) receiving neuropathologic diagnoses of AD. Five cases had study classifications of probable MCI-LB, with all 5 (100%) having DLB confirmed at autopsy: 1 had pure DLB (neocortical LBD with low AD related neuropathologic changes), 2 had neocortical LBD with intermediate AD related changes, and 2 had neocortical LBD with high AD related changes. One case who had been excluded prospectively because of suspected vascular cognitive impairment at baseline also had this diagnosis confirmed at subsequent autopsy.

All 5 autopsy LBD cases (100%) had clinically progressed to DLB before death as confirmed by either study or brain bank records. Three of 4 (75%) pathologic cases of AD remained mildly impaired with clinical diagnosis of MCI-AD at the time of death, with 1 (25%) having been diagnosed with dementia due to AD.

## Discussion

We prospectively assessed a cohort of people with MCI differentially classified as MCI-AD or MCI-LB at baseline, examining specifically their diagnostic stability and rates of progression to dementia and death.

We found that MCI-LB was a stable and reliable diagnosis, which converted to DLB at high rates over 3–5 years. MCI-AD had similar rates of conversion to dementia with good diagnostic stability.

Overall rates of diagnostic revision were relatively low, with most cases of probable DLB categorized as probable MCI-LB at baseline. All probable MCI-LB who converted to dementia were seen to develop DLB.

These findings are evidence that the previously published research criteria for diagnosis of MCI-LB have prospective validity and so are suitable for use in clinical practice^2^ and add to a growing evidence base demonstrating the importance of recognizing this clinical syndrome within the broader MCI umbrella.

We previously reported accelerated dementia onset over the short term (1–2 years) in MCI-LB in a subset of this cohort.^9^ We find here that this does not necessarily translate into greater dementia rates overall in MCI-LB than MCI-AD over the medium term. An exploratory analysis accounting for possible diagnostic group-gender interactions provided some tentative support for greater dementia rates in MCI-LB. However, any quantitative comparisons are necessarily limited by the relatively low numbers overall and high rates of dementia in both groups with few remaining as MCI by 5 years.

Death is a competing risk for the development of dementia. Rates of clinical progression may be underestimated when considering observed dementia onset alone. This is a particular concern in conditions such as DLB, which are known to have poorer survival than AD,^25^ and have been reported to have a particularly rapid decline in some cases.^26^ Such rapid decline might cause dementia to be missed between the last research observation and death. We, therefore, examined death as a competing adverse outcome in these cohorts and, where available, examined linked brain bank findings to determine whether cases died with dementia. Brain bank records indicated that MCI-LB cases who died before dementia being seen in study likely had DLB at the time of death, agreeing with previous findings demonstrating that deaths in synucleinopathies are most frequently primarily attributable to the neurodegenerative process.^27^ This was not necessarily the case in MCI-AD, who often died while still reported to be mildly impaired. Dementia incidence may, therefore, be slightly underestimated in the MCI-LB group.

The rates of dementia in this sample are higher than often seen in research settings but may be more representative of the rates of dementia in MCI due to a progressive neurologic disease. Similarly aged population studies have described MCI to dementia progression rates as low as 11% over a comparable time period.^28^ While specialist settings typically report higher rates of dementia progression than community/population settings, most research participants with MCI do not develop dementia over this time period.^29^ Progression rates in this study approximate or exceed the crude annualized progression estimates reported in previous studies without comprehensive phenotyping,^30^ and 3-year progression rates are comparable with studies with comprehensive AD biomarker risk stratification.^31^

In this cohort, participants with MCI had a relatively low average level of cognitive performance possibly being closer to the dementia threshold according to ACE-R scores initially and were also required to meet disease-specific criteria for MCI-AD or MCI-LB for inclusion. This lower baseline and careful assessment to identify these specific etiologies may have reduced the prevalence of nonprogressive MCI/dementia mimics (e.g., mood disorders and functional cognitive disorders) in comparison with other MCI subtyping methods (e.g., any-cause MCI, or neuropsychological cutoffs). To limit potential bias, participants later believed to have a subjective cognitive impairment only were retained in their initial classification groups for analysis.

Participants who withdrew or were lost to follow-up have an unknown outcome and so were excluded from primary analysis. We sought additional information from clinical records or brain banks where available, with this information incorporated in the primary analysis. In a sensitivity analysis, we examined the impact of carrying the last observation forward for participants with an entirely unknown outcome, conservatively assuming that they remained as MCI indefinitely. This did not meaningfully change the results, with high rates of MCI progression at 3 and 5 years in both groups, albeit slightly attenuated in comparison with the primary analysis. These sensitivity estimates are likely to be highly conservative, however. Study withdrawal or loss to follow-up may itself reflect unseen clinical progression as greater cognitive impairment is associated with lower research participant retention.^32^ However, in this cohort, those lost to follow-up did not appear to have a better or worse level of baseline function or age/education-related risk than those with follow-up data available.

Amyloid PET or CSF markers were not available for the confirmation of AD in this cohort. Although numbers were limited, there were several cases with neuropathologic assessment available. This represents the diagnostic gold standard and indicated 100% accuracy to date for baseline diagnosis by the clinical panel. As noted above, the rate of conversion from MCI-AD to AD was relatively high, supporting the likely presence of AD pathophysiologic changes or some other progressive pathology, in this group. Diagnosis of MCI-AD and AD in this cohort remains one of exclusion and other pathologic changes (e.g., vascular cognitive impairments) could be present in these cases, although we think this is unlikely because those with evidence of stroke disease or significant cerebrovascular disease on neuroimaging were excluded at baseline and follow-up reviews.

Diagnosis of MCI-LB or DLB does not necessarily indicate absence of AD copathology, which is known to be common, and has been confirmed in 4 of 5 DLB cases who have undergone autopsy to date. AD copathology may have implications for the progression of MCI-LB and should be examined in future work as more cases come to autopsy.

These findings support that prospectively identified MCI-LB is a prodromal manifestation of DLB. MCI-LB typically converts to dementia within 3–5 years and does so at very high rates.

## Appendix Authors

**Table:**

Name	Location	Contribution
Calum A. Hamilton, PhD	Translational and Clinical Research Institute, Newcastle University, United Kingdom	Drafting/revision of the manuscript for content, including medical writing for content; major role in the acquisition of data; study concept or design; analysis or interpretation of data
Paul C. Donaghy, PhD	Translational and Clinical Research Institute, Newcastle University, United Kingdom	Drafting/revision of the manuscript for content, including medical writing for content; major role in the acquisition of data; study concept or design
Rory Durcan, PhD	Translational and Clinical Research Institute, Newcastle University, United Kingdom	Drafting/revision of the manuscript for content, including medical writing for content; major role in the acquisition of data
Joanna Ciafone, PhD	Translational and Clinical Research Institute, Newcastle University, United Kingdom	Drafting/revision of the manuscript for content, including medical writing for content; major role in the acquisition of data
Kirsty Olsen, MSc	Translational and Clinical Research Institute, Newcastle University, United Kingdom	Drafting/revision of the manuscript for content, including medical writing for content; major role in the acquisition of data
Gemma Roberts, PhD	Translational and Clinical Research Institute, Newcastle University, United Kingdom	Drafting/revision of the manuscript for content, including medical writing for content; major role in the acquisition of data
Michael J. Firbank, PhD	Translational and Clinical Research Institute, Newcastle University, United Kingdom	Drafting/revision of the manuscript for content, including medical writing for content; major role in the acquisition of data
Louise M. Allan, PhD	Centre for Research in Ageing and Cognitive Health, University of Exeter, United Kingdom	Drafting/revision of the manuscript for content, including medical writing for content; study concept or design
John-Paul Taylor, PhD	Translational and Clinical Research Institute, Newcastle University, United Kingdom	Drafting/revision of the manuscript for content, including medical writing for content; major role in the acquisition of data; study concept or design
John T. O'Brien, DM	Department of Psychiatry, School of Clinical Medicine, University of Cambridge, United Kingdom	Drafting/revision of the manuscript for content, including medical writing for content; study concept or design
Alan J. Thomas, PhD	Translational and Clinical Research Institute, Newcastle University, United Kingdom	Drafting/revision of the manuscript for content, including medical writing for content; major role in the acquisition of data; study concept or design

## Glossary

ACE-R: Addenbrooke's Cognitive Examination—Revised
AD: Alzheimer disease
DLB: Dementia with Lewy bodies
FP-CIT: 123I-N-ω-fluoropropyl-2β-carbomethoxy-3β-(4-iodophenyl)nortropane
FTD: frontotemporal dementia
HMR: heart to mediastinum MIBG uptake ratio
MCI: mild cognitive impairment
MCI-AD: MCI due to AD
MCI-LB: MCI with Lewy bodies
MIBG: 123I-metaiodobenzylguanidine
NIA-AA: National Institute on Aging-Alzheimer's Association
OR: odds ratio
PSP: progressive supranuclear palsy
SCI: subjective-only impairment
UPDRS-III: Unified Parkinson's Disease Rating Scale—Motor Examination
VCI: vascular cognitive impairment
